# Land use/land cover changes due to gold mining in the Singida region, central Tanzania: environmental and socio-economic implications

**DOI:** 10.1007/s10661-025-13921-x

**Published:** 2025-03-25

**Authors:** Azaria Stephano Lameck, Brian Rotich, Abdalrahman Ahmed, Harison Kipkulei, Silvester Raymond Mnyawi, Kornel Czimber

**Affiliations:** 1 Doctoral School of Environmental Science, The Hungarian University of Agriculture and Life Sciences, Páter Károly U. 1, Gödöllő, 2100 Hungary; 2 Department of Earth Science, Mbeya University of Science and Technology, PO BOX 131, Mbeya, Tanzania; 3 Faculty of Environmental Studies and Resources Development, Chuka University, P.O. Box 109-60400, Chuka, Kenya; 4 Institute of Geomatics and Civil Engineering, Faculty of Forestry, University of Sopron, Bajcsy-Zs 4, Sopron, 9400 Hungary; 5 Department of Forest and Environment, Faculty of Forest Science and Technology, University of Gezira, Wad Medani, Sudan; 6 Institute of Geography, Faculty of Applied Computer Sciences, University of Augsburg, Alter Postweg 118, 86159 Augsburg, Germany; 7 Department of Natural Science, Mbeya University of Science and Technology, PO BOX 131, Mbeya, Tanzania

**Keywords:** LULC change, Mining sites, Gold rush miner, Environmental implications, Social-economic implications

## Abstract

This study explored the land use and land cover (LULC) changes (1995–2023) in the gold mining hotspots of Mang’onyi, Sambaru, and Londoni in the Singida region of Tanzania. The study integrated remote sensing (RS) to evaluate the LULC transitions with social survey assessments (83 respondents) to determine the resident’s perceptions of the environmental, social, and economic implications of mining bridging technical data with socio-economic realities. Supervised classification of Landsat images was conducted using the random forest (RF) classifier to generate LULC maps with five classes (bareland, agricultural land, forest, built-up, and shrubs and grasses), followed by an analysis to identify LULC change trends. The results showed an overall increase in agricultural land 168.51 km^2^ (587.55%), bareland 7.70 km^2^ (121.45%), and built-up areas 0.55 km^2^ (134.15%), while forest and shrubs and grasses areas declined by 97.67 km^2^ (− 72.59%) and 79.09 km^2^ (− 43.49%), respectively. A social survey assessment revealed residents perceived environmental (deforestation, biodiversity loss, land degradation, water, air, soil pollution), social (occupational hazards, land use conflicts, negative effects on livelihoods and culture, discrimination, child labor, community displacement), and economic (improved housing, infrastructural development, job creation, economy boost, improved access to services) impacts resulting from mining activities. Our findings underscore the importance of balancing the economic benefits of gold mining with the imperative to protect the environment and support sustainable livelihoods in the mining regions.

## Introduction

Mining has been a key player in most nations economic development, infrastructural development, employment, and supply of essential raw materials (Worlanyo & Jiangfeng, 2021). It has served as a viable route to economic transformation and industrialization in resource-rich developed countries and is an equally significant component in many low- and middle-income nations since mineral resources are necessary for the development of their economies (Ericsson & Löf, 2019; Kuzevic et al., 2022). Many low-income sub-Saharan Africa (SSA) countries are large producers and exporters of lucrative minerals such as diamonds, crude oil, bauxite, gold, chromite, platinum, cobalt, titanium, and rare earth elements (Pokorny et al., 2019). Social and economic development indicators have shown signs of progress for African countries that are rich in mineral resources (Ericsson & Löf, 2019). It is evident that mining operations and exports have played a pivotal role in the economies of SSA countries like the Democratic Republic of Congo (DRC), Malawi, Ghana, South Africa, and Guinea. It is reported that 25% of Guinea’s and 5.9% of South Africa’s gross domestic profits (GDP) as well as the majority of their foreign revenues are mining-related (Aryee, 2001). Mineral exports constitute 86% of total exports in the DRC and contribute to 13% of the country’s GDP (Ericsson & Löf, 2019). In Ghana, gold mining contributes about 5.7% of the national GDP (Mensah et al., 2015), while uranium mining operations in Malawi spurred the contribution of the mining sector to 10% of the GDP in 2010 from less than 3% in 2005 (Haundi et al., 2021). While it is evident that mining has transformed many economies, it has also had negative impacts on the environment and society (Worlanyo & Jiangfeng, 2021). The extraction of minerals, especially through opencast mining, impacts the land, soil, water, and vegetation. Artisanal and small-scale mining is associated with several negative impacts, including, loss of mineral revenue due to smuggling, food insecurity, contamination and pollution of surface and underground water sources, air and noise pollution, and biodiversity loss (loss of natural flora and fauna) (Gbedzi et al., 2022; Suglo et al., 2021; Yu & Zahidi, 2023). A notable example is the increasingly alarming levels of water pollution caused by illegal artisanal and small-scale mining (Galamsey) ‘Menace’ in Ghana, which have sparked serious debate among policymakers, environmentalists, and local communities (Eduful et al., 2020; Kuffour et al., 2018; Suglo et al., 2021). Recently, some illegal miners were holed up in the vast tunnel network of the Stilfontein mine in South Africa. Negative health risks associated with illegal mining include fatal accidents, injuries, respiratory and skin diseases, noise-induced hearing loss, physical and psychological stress, malaria, HIV, and other infectious diseases (Al Rawashdeh et al., 2016; Suglo et al., 2021; Worlanyo & Jiangfeng, 2021).

Tanzania is rich in minerals like diamond, gold, bauxite, Tanzanite, limestone, tin, and copper, contributing significantly to its economy (Yager, 2023). The country is the third-largest gold producer in Africa, after South Africa and Ghana, accounting for about 1% of the world’s gold output in 2019 (Lugoe, 2011; Yager, 2023). Gold mining, driven by sector liberalization and high gold prices, has become a major economic contributor, representing over 41.3% of export earnings and 3.6% of GDP (Lugoe, 2011). In 2017, Tanzania’s large-scale mines employed around 12,000 workers, with an estimated 1.5 million artisanal miners also involved (Yager, 2023). Gold accounts for more than 41.3% of Tanzania’s export earnings, 75% of foreign direct investment (FDI), and an increasing share of taxes, representing 3.6% of the gross domestic product (GDP) (Lugoe, 2011). The liberalization and privatization of the mining sector and the soaring gold prices during the last decades have made gold mining increasingly attractive and triggered a gold boom in Tanzania (Hammond et al., 2007; Lugoe, 2011; Phillips et al., 2001).

The Singida region, known for its gold reserves, has been a mining site since 1909, with notable sites like Sekenke, Shelui, and Muhentiri. Discoveries in Londoni, Sambaru, and Mang’onyi in 2004 further highlight its gold potential. Mining became semi-mechanized (modern equipment) in the early 2000s, leading to environmental and social changes in local communities (Herman & Kihampa, 2015; Lugoe, 2011). Shanta Gold Mine Company, which began surveying in 2004, achieved commercial production in 2023, reflecting the region’s growing mining industry (Shanta Gold Limited, 2024). Mining in the area includes local artisanal miners, gold rush miners, and small-scale companies, whose influx has significantly impacted the environment, local economy, and social dynamics (Jønsson & Bryceson, 2009; Poignant, 2023). Assessment of the cumulative environmental impacts of mining is an important aspect of sustainable management as it involves balancing the benefits of resource exploitation against environmental degradation (Latifovic et al., 2005). The conflict between mining and environmental protection has intensified, highlighting the need for better information on mining impacts at regional and local levels (Latifovic et al., 2005). Therefore, it is essential to map and monitor the impacts of gold mining on the LULC dynamics in the areas surrounding the mining sites of Mang'onyi, Sambaru, and Londoni in the Ikungi and Manyoni districts of the Singida region, Tanzania.

This study aims to (a) assess the changes in LULC dynamics (1995–2023) due to gold mining activities and (b) examine the societal perceived environmental and socio-economic implications of these LULC changes. The mapping of LULC dynamics and the subsequent analysis to identify change trends at the study site provide comprehensive evaluations of mining-induced alterations, offering valuable new insights into the environmental impacts of mining activities in the area. Integration of remote sensing (RS) data with local community perceptions of the environmental, social, and economic effects of mining further strengthens the study’s originality, bridging technical data with socio-economic realities. The study provide a comprehensive analysis of the extent of LULC dynamics and community perceptions regarding the environmental and socio-economic impacts of mining activities in the study site. The study also provide valuable practical insights for policymakers, stakeholders, and local communities aiming to promote sustainable mining practices that balance environmental protection, economic growth, and the livelihoods of the local communities in mining sites. These insights will help strengthen the enforcement of environmental regulations at mining sites. This, in turn, will ensure that mining practices are better regulated, minimizing adverse impacts on both the environment and local communities.

## Materials and methods

### Description of the study area

**Fig. 1:**
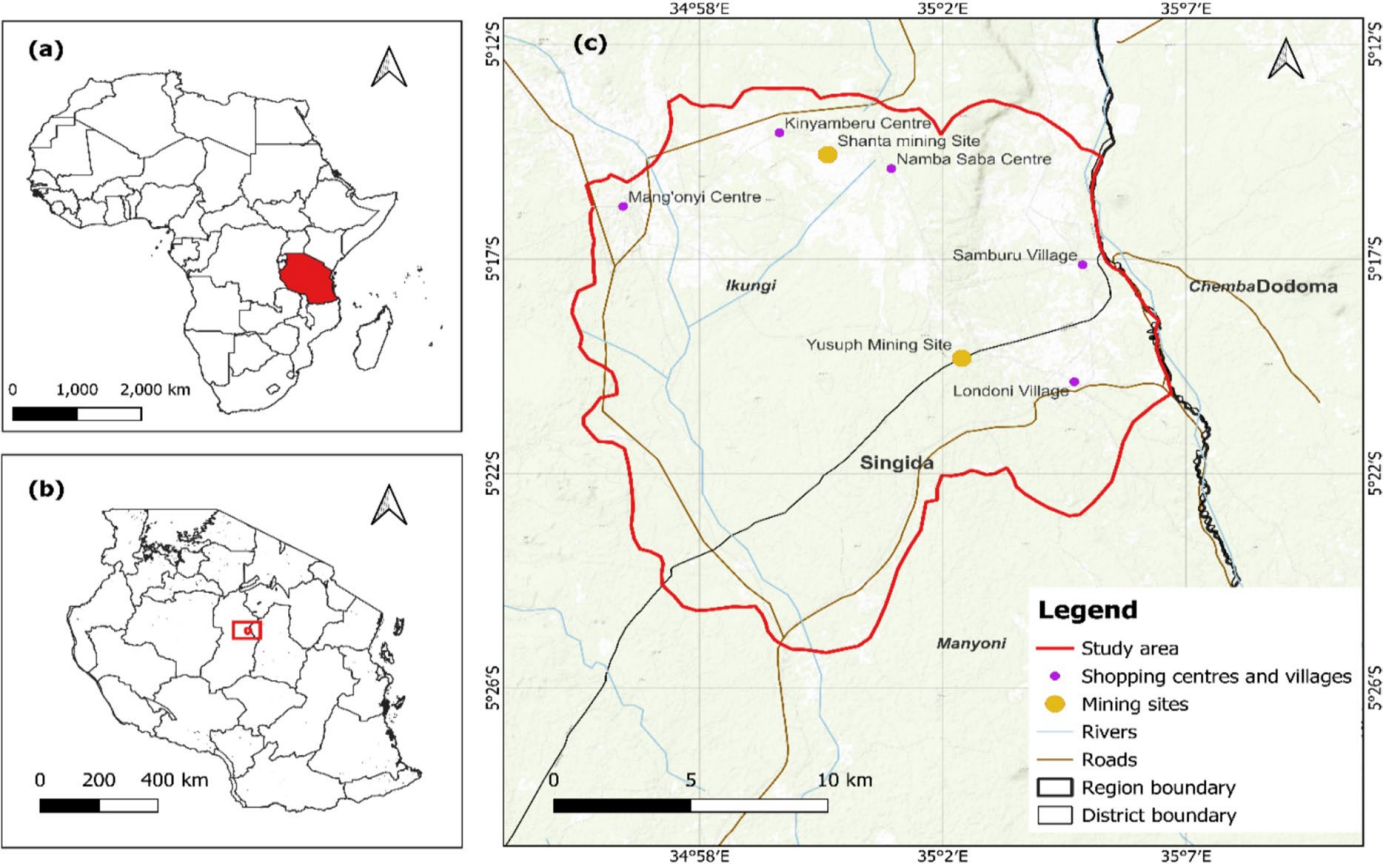
Map of the study area showing (**a**) the location of Tanzania in Africa, (**b**) the location of the study area in the Singida region of Tanzania, and (**c**) the mining sites, villages, and administrative boundaries in the study area

**Fig. 2:**
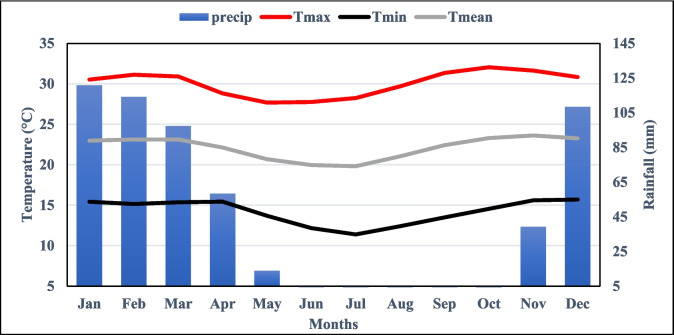
The mean annual precipitation and temperature (*T*_max_, *T*_min_, and *T*_mean_) of the study area

This study covers three villages (Mang’onyi, Sambaru, and Londoni) situated on the border of the Ikungi and Manyoni districts in the Singida region of Tanzania (Fig. 1). These villages are located within longitudes 34°54′ and 35°7′E and latitudes 5°12′ and 5°26′S, covering an estimated area of 351.82 km^2^. The region experiences a semi-arid climate, characterized by a rainy season from late November to early May and a prolonged dry season from June to early November (Fig. 2). Annual precipitation ranges between 400 and 600 mm, while temperatures vary from 14 to 30 °C, with a mean annual temperature of 22 °C (Fig. 2). The local population depends largely on agriculture, cultivating maize, sorghum, and sunflowers while also keeping livestock such as goats, sheep, and cattle. However, after discovering gold deposits in 2004, villagers started engaging in small-scale mining as an additional source of income. The mining operations are conducted by a legally independent multinational company that has obtained a permit from the Ministry of Minerals and Energy. However, there are a reasonable number of small miners (artisans) who illegally exploit minerals to sustain their livelihoods. In Londoni village, gold mineral deposits were first discovered in June 2004 by Mr. Jumanne Mtemi, followed by active mining activities from July 2004 (Jønsson & Bryceson, 2009). This accelerated the exploration and discovery of more gold reserves (Mustapha, Marando, and Yusuph mining sites) in Sambaru village from September to October 2004. Other gold deposits, including Mwau (Hanje), Shanta Number 1, and the Taru Shanta mining site, were discovered in the subsequent years. The mining sites particularly in Londoni and Sambaru are located on a steep ridge in the Rift Valley, with reddish iron-rich soils, thorny plants, grasses, and tiny trees (Herman & Kihampa, 2015). In the region, gold extraction involves crushing, grinding ore, washing, and capturing gold particles using sluice tables, amalgamation, burning, and cyanide solution leaching of tailings (Herman & Kihampa, 2015). In Mang’onyi village, particularly in Kinyamberu and Taru, the Shanta Gold Mine Company began a survey in 2004, subsequently initiating the construction of the mining site in late 2020. Shanta Gold Mine Company commenced mining operations in September 2021, with ore stockpiled, and reached commercial production in 2023 (Shanta Gold Limited, 2024).

### Methodology

#### Data acquisition

Landsat 5 and 8 Surface Reflectance (SR) data, with a spatial resolution of 30 m, were acquired from the Google Earth Engine (GEE) platform due to their accessibility and the platform’s capability for preliminary data processing. We utilized the median surface reflectance values of Landsat 5 and 8 imageries from June 1995, 2004, 2014, and 2023, corresponding to the study area’s driest period. This approach minimizes cloud cover and ensures comprehensive spatial coverage and temporal consistency. The year 1995 was selected for comparison as it represents the period before the discovery of mining in 2004 (Lugoe, 2011; United Republic of Tanzania, 2014), while the years 2014 and 2023 were chosen to assess changes that occurred after the discovery of gold mining.

#### Training and testing sample datasets

Multi-temporal Google Earth aerial imagery and various color composites of different bands (SR_B1, SR_B2, SR_B3, SR_B4, SR_B5, SR_B6, SR_B7) of Landsat data were utilized to identify appropriate training sites. Training and testing samples were collected through on-screen digitizing, as widely used and reported in the literature (Ahmed et al., 2024). Furthermore, the identification of the training sites was corroborated with high-resolution Google Earth imagery. Additionally, the first author’s extensive knowledge of the study area was instrumental in accurately determining the ground truthing for spotting land cover classes. The QGIS 3.34.3 software was employed to generate training and testing sample data for each land cover class. A total of 148, 140, 144, and 150 training samples were created for the years 1995, 2004, 2014, and 2023, respectively. These samples were randomly divided, with 50% allocated for training and 50% for accuracy validation. In supervised satellite image classification, the typical data split for training and testing is 70–30% or 80–20%. However, some studies have successfully used a 50–50% split achieving overall accuracy of between 81 and 93% (Ahmed et al., 2024; Stephano et al., 2025). This approach provides a better estimate of generalization performance, reducing the risk of overfitting the training data and ensuring that results are not inflated due to insufficient test data (Yadav et al., 2024).

#### Image classification

Supervised classification was performed using the Dzetsaka plugin in QGIS, which supports various machine-learning algorithms. To leverage Dzetsaka’s capabilities, additional dependencies, including the Scikit-learn 1.0.1 Python package—a standard library for machine learning (Karasiak, 2020; Pedregosa et al., 2011), were installed. The random forest (RF) classifier was utilized to classify the three Landsat images due to its robustness in distinguishing various LULC classes across different environmental areas (Rodriguez-Galiano et al., 2012; Thonfeld et al., 2020) and its high accuracy in this study. The RF algorithm, a decision-tree-based ensemble learning method, has been employed to address various environmental problems and can process a wide range of data, including satellite images and numerical data (Oo et al., 2022). The algorithm also has several other advantages including the ability to model non-linear relations, ability to handle outliers, and sensitivity to overfitting. This study used the Dzetsaka classification tool in QGIS to apply RF classification to the Landsat images. A fixed number of 100 trees was set by default, which is an appropriate size to avoid overfitting (Breiman, 2001; Rubo et al., 2019). Five classes of LULC, including forestland, shrubs and grasses, agricultural land, bareland, and built-up areas, were successfully classified. In this study, mining areas were not classified as a distinct land cover type due to the complex nature of mining activities in the region. Legal mining sites are often visible as bare land in remote sensing imagery, while illegal mining occurs within shrublands and grasslands, making it difficult to delineate separately. The spectral similarity between mining areas and bareland, along with the dynamic nature of mining activities (where abandoned sites may undergo natural revegetation), posed classification challenges. To account for mining-induced land cover changes, we analyzed transitions in bare lands, which are closely linked to mining expansion. This approach has similarly been used by other researchers in classification (Gbedzi et al., 2022). Field observations, expert knowledge, and Google Earth imagery were additionally used to validate mining-related changes. This approach ensured that both legal and illegal mining impacts were captured while minimizing classification bias.

#### Accuracy assessment

Accuracy assessment is a critical step in the classification process, aimed at quantitatively evaluating the effectiveness of pixel classification into the correct land cover classes. Following the successful classification of LULC, the Semi-automatic Classification Plugin (SCP) in QGIS was utilized to assess the accuracy of the classified maps. A random stratified sample, with an equal number of samples for each land cover class, was employed to train and test the classifier by computing several metrics and presenting them using the confusion matrix (Ahmed et al., 2024). The confusion matrix is the most common method for presenting the accuracy of classified images (Plourde & Congalton, 2003). Other accuracies included are the overall accuracy and the kappa index. The kappa coefficient ranges from negative values to 1, where 0 indicates no agreement and 1 indicates perfect agreement. A kappa value around zero suggests a fully random classification, while negative values indicate a classification worse than random ( Landis & Koch, 1977a, 1977b).

#### Intensity analysis and LULC transitions

LULC dynamics in the study region were analyzed using intensity analysis. The analysis decomposes changes into interval, category, and transition levels. The analysis incorporates a mathematical framework that compares a uniform intensity to the observed temporal changes among various land use categories. Uniform intensity is a hypothetical intensity where the overall change during the time interval was uniformly distributed across the classes. Intensity analysis helps determine the spatial distribution and magnitude of landscape changes by identifying land use categories that exhibit the most substantial shifts compared to the uniform rate of change. It provides information about the rate and extent of land cover changes over time. It measures the degree to which these changes are non-uniform, highlighting which land categories are most active or relatively dormant within a given time interval. The technique uses cross-tabulation matrices, where each matrix summarizes the LULC change at each time interval.

The intensity analysis implements this in three levels: the interval level, the category level, and the intensity level. The interval level analyzes the overall change size and annual change intensity for the whole area in each time interval. The annual change intensity of the study area during time *t*(*S*_*t*_) is given by:

1 $${S}_{t}= \frac{\sum_{j}^{I}[(\sum_{i=1}^{J}{C}_{tij})-{C}_{tij}}{({Y}_{t+1}-{Y}_{t})(\sum_{j}^{I}\sum_{i=1}^{J}{C}_{tij})}$$

where *S*_*t*_ is the annual change intensity, *J* is the number of categories, *j* is the index of the category at a later time point, and *i* is the index of the category at an initial time point. *C*_*tij*_ is the size of the transition from category *i* to category *j* during the interval *Y*_*t*_ and *Y*_*t* +1_. *Y*_*t*_ is the year at time point *t*, and *Y*_*t* +1_ is the year at time point *t* + 1. The upper part of Eq. 1 gives the change during the time interval *t*, while the lower part is the product of the study area size and the interval duration.

Uniform change intensity (*U*_*t*_) during interval *t* is shown in the following equation:

2 $${U}_{t}=\frac{\sum_{t=1}^{T-1}(\sum_{j=1}^{J}\sum_{j=1}^{J}{C}_{tij})}{{(Y}_{T}-{Y}_{1})(\sum_{j}^{J}\sum_{i=1}^{J}{C}_{tij})} \times 100$$

The upper part of the equation computes the change during all intervals, and the denominator gives the product of the study area size and the study duration.

The category level provides information on the variation in size and intensity of gross gains and losses across categories during each period. Therefore, loss intensity (Altieri et al., 2015) from category *i* during the time interval *t* corresponds to the percentage lost at the beginning of the time interval t (Eq. 3) (Allaire et al., 2022). On the other hand, the gain intensity (*G*_*tj*_) is the percentage of the end size of category *j* gained during the time interval *t* (Eq. 4). If *L*_*ti*_ < *S*_*t*_ or *G*_*tj*_ < *S*_*t*_, then the loss from category *i* or gain to category *j* during the time interval *t* is dormant. The reverse indicates activeness between the categories in the time interval *t*.

3 $${L}_{ti}=\frac{\sum_{i=1}^{J}{C}_{tij}-{C}_{tii}}{({Y}_{t+1}-{Y}_{t}){\sum }_{i=1}^{J}{C}_{tij}}*100$$

4 $${G}_{tj}=\frac{\sum_{i=1}^{J}{C}_{tij}-{C}_{tjj}}{({Y}_{t+1}-{Y}_{t}){\sum }_{i=1}^{J}{C}_{tij}}*100$$

The intensity analysis was implemented using the OpenLand package in the R statistical software (Exavier & Zeilhofer, 2020). The package uses the LULC classifications in the studied periods as input data in the contingency table to generate a quantity of change from one category to another between two points. The changes between various class categories were also represented using a Sankey plot. The Sankey plot visualizes flows through a network. It incorporates the cross-tabulation matrices, which contain information on the sizes of categorical differences between two maps to describe the amount and type of land cover change that has occurred between two points in time.

### Perceived implications of gold mining

A social survey was conducted from 2nd to 28th of September 2024 to support LULC change results from the satellite data. Household (HH) questionnaires with open and close-ended questions were administered to the residents using the ODK collect application to solicit information. The questions focused on household demographics, types of mining, previous LULC types, and the impacts (environmental, economic, and social) of mining activities in the study area. Stratified sampling was used to distribute the questionnaires among 83 households in the three mining villages. Key informant interviews (Gbegbelegbe et al., 2018) were also conducted with environmental officers, community elders, mining companies, and non-governmental organizations (Pinto et al., 2013) in the study area to capture information that might have been overlooked in the HH questionnaires and simultaneously enrich information gathered through HH surveys. Field observations were also made at the mining sites during the surveys.

## Results

### Accuracy result table

Land use classification revealed high accuracies (Table 1). The overall accuracies ranged between 80 and 88%, while the kappa index ranged between 0.68 and 0.82 for the studied years. Since these results fell within the acceptable range, we proceeded with utilizing the classification outputs (Ahmed et al., 2024; Landis & Koch, 1977a, 1977b; Patil & Nataraja, 2020).

**Table 1:** LULC classification accuracies

	1995	2004	2014	2023
Overall accuracy	80.96	88.41	81.77	80.59
Kappa hat	0.68	0.82	0.72	0.69

### Distributions of different LULC classes and statistics

**Fig. 3:**
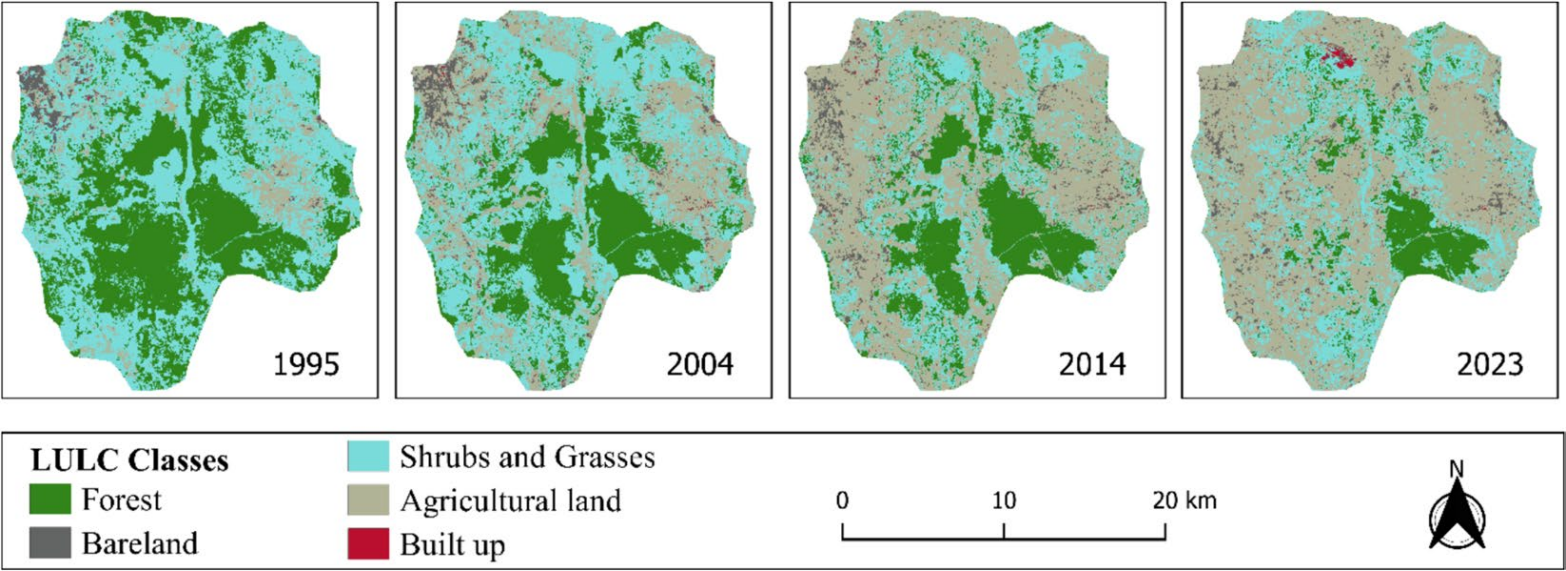
LULC class coverage in the study area in 1995, 2004, 2014, and 2023

The land cover statistics and the land cover maps showing the distribution of the different LULC classes across the study area are presented in Table 2 and Fig. 3. Statistical analysis shows that in 1995, shrubs and grasses were the dominant LULC class at 181.84 km^2^ (51.68%) while forest had the second largest coverage of 134.55 km^2^ (38.25%). Built-up areas had the least area coverage at 0.41 (0.12%) (Table 2). In 2004, shrubs and grasses still covered most parts of the study area at 161.50 km^2^ (45.90%), agricultural land had the second largest area coverage of 90.42 km^2^ (25.70%), while forest had the third largest area at 89.44 km^2^ (25.42%) (Table 2). Bareland had the second least area of 9.87 km^2^ (2.81%), while built-up areas occupied the least area of our study region at 0.59 km^2^ (0.17%). By 2014, agricultural land had dominated the study area with a coverage of 178.46 km^2^ (50.73%) over shrubs and grasses, with the second largest area at 86.74 km^2^ (24.65%). Forest came in third with a reduced area of 73.85 km^2^ (20.99%). Despite increasing size, built-up areas still had the smallest area coverage of 0.66 km^2^ (0.19%). In 2023, agricultural land coverage increased to 197.19 km^2^ (56.05%), shrubs and grasses covered 102.75 km^2^ (29.21%), forest 36.88 km^2^ (10.48%), bareland 14.04 km^2^ (3.99%), and built up made up 0.96 km^2^ (0.27%) of the study area (Table 2).

**Table 2:** Land use and land cover statistics

LULC class	1995 (km^2^)	%	2004 (km^2^)	%	2014 (km^2^)	%	2023 (km^2^)	%
Forest	134.55	38.25	89.44	25.42	73.85	20.99	36.88	10.48
Bareland	6.34	1.80	9.87	2.81	12.11	3.44	14.04	3.99
Shrubs and grasses	181.84	51.68	161.50	45.90	86.74	24.65	102.75	29.21
Agricultural land	28.68	8.15	90.42	25.70	178.46	50.73	197.19	56.05
Built up	0.41	0.12	0.59	0.17	0.66	0.19	0.96	0.27
Total	351.82	100.00	351.82	100	351.82	100	351.82	100

**Fig. 4:**
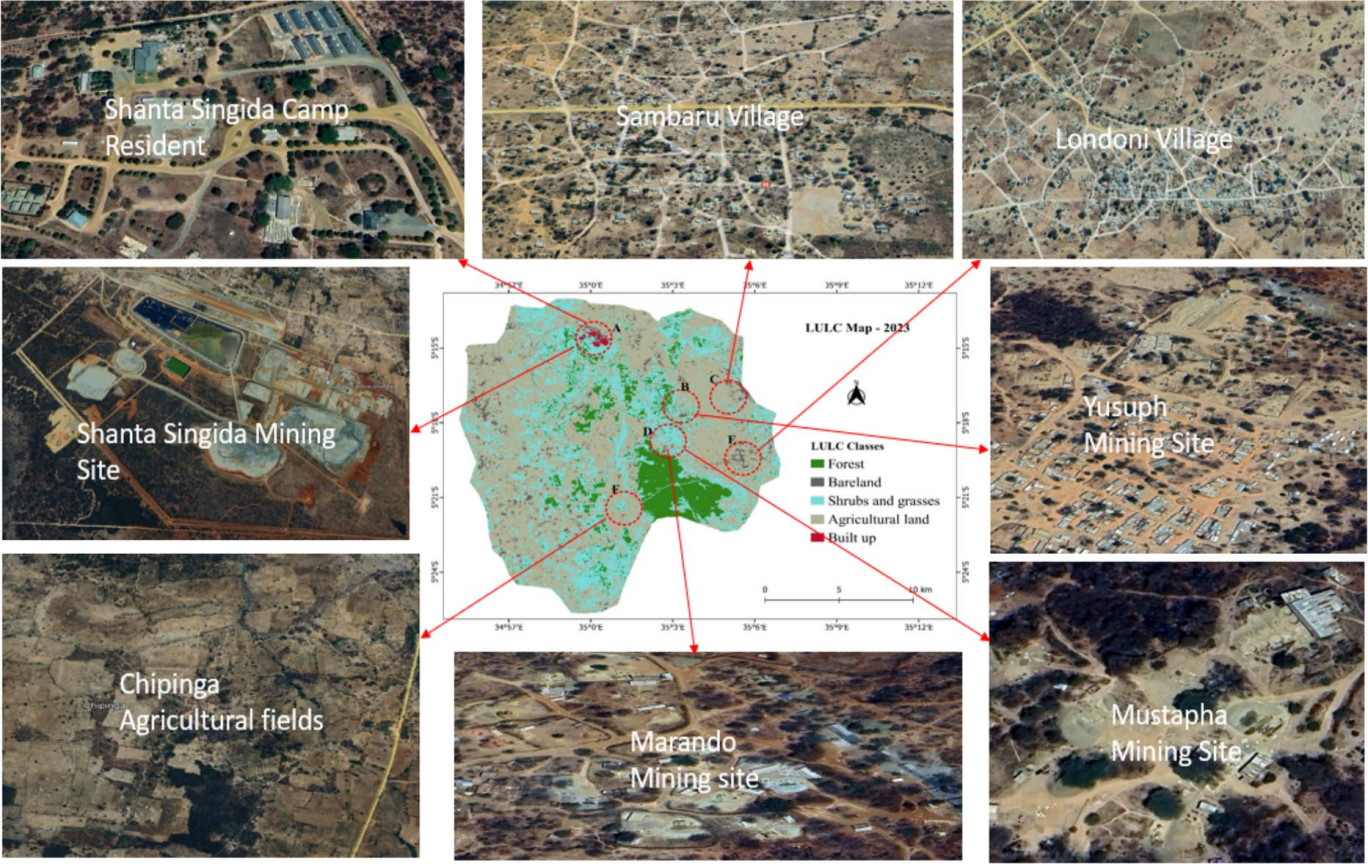
The LULC map of 2023 shows active mining sites and the study area villages

From the maps (Fig. 3), the dominant LULC classes in the study area were agricultural lands, shrubs and grasses, and forest lands. Forested areas dominating in the central and southern parts of the study and declined over time notably the area around Chipinga, with agricultural areas, shrublands, and bareland areas occupying formerly forested areas. There was a continuous expansion of agricultural areas, with grasslands and shrublands contributing much of this transformation. Bareland areas in the northwestern part of the study area around the Mang’onyi trading centre slowly diminished, with much of the area being occupied by agricultural land. Bareland areas also expanded, especially in the eastern parts of the study area where most of the mining sites (Yusuph, Marando) and villages (Londoni, Sambaru) are located (Fig. 4). There was a surge in built areas between 2014 and 2023 notably around the Shanta Singida mining site in the northern part of the study area (Figs. 3 and 4).

### The area changes of the different LULC classes

**Fig. 5:**
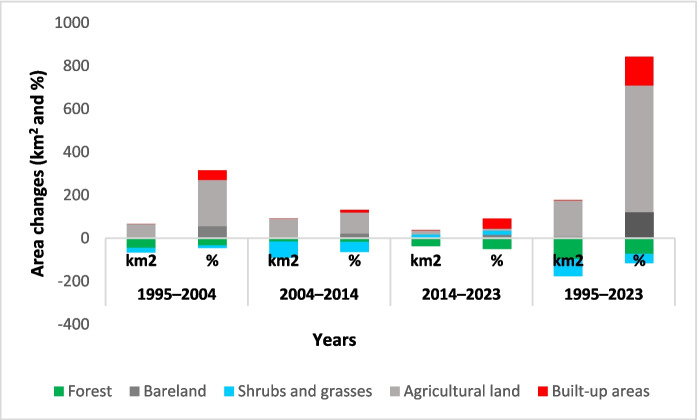
LULC class change (1995–2004, 2004–2014, 2014–2023, and 1995–2023)

The study area underwent significant transformations in its major LULC categories, displaying diverse patterns and varying degrees of change (Fig. 5). The first study period (1995–2004) experienced an expansion in agricultural land, bareland, and built-up areas by 61.74 km^2^ (215.27%), 3.53 km^2^ (55.68%), and 0.18 km^2^ (43.90%), respectively. In the second decade (2004–2014), agricultural land increased significantly by 88.04 km^2^ (97.37%). Similarly, bareland increased by 2.24 km^2^ (22.70%) and built-up areas by 0.07 km^2^ (11.86%). On the other hand, shrubs and grasses declined by 74.76 km^2^ (− 46.29%) and forests by 15.59 km^2^ (− 17.43%). In the third period (2014–2023), agricultural land, shrubs and grasses, bareland, and built-up areas all increased by 18.73 km^2^, 16.01 km^2^, 1.93 km^2^, and 0.3 km^2^, respectively, while forest areas diminished by 36.97 km^2^. Overall (1995–2023), there was an increase in agricultural land 168.51 km^2^ (587.55%), bareland 7.70 km^2^ (121.45%), and built-up areas 0.55 km^2^ (134.15%), while forest and shrubs and grasses areas declined by 97.67 km^2^ (− 72.59%) and 79.09 km^2^ (− 43.49%), respectively (Fig. 5).

### LULC change intensity analysis

**Fig. 6:**
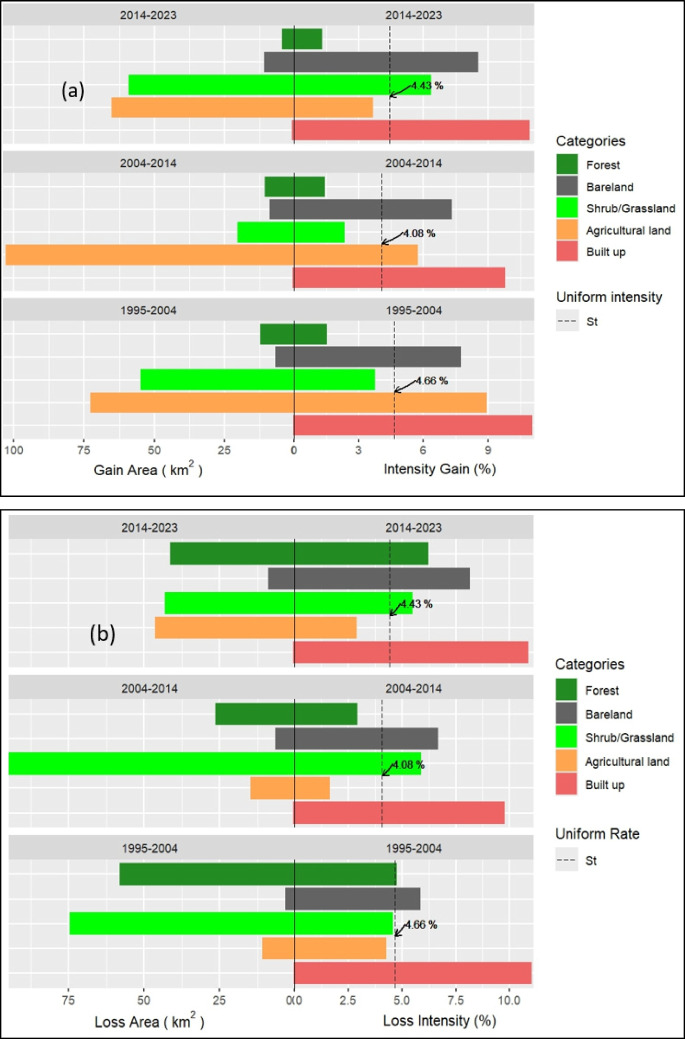
Category intensity analysis. **a** Intensity gain area outcomes and **b** intensity loss area outcomes for three-time intervals: 1995–2004, 2004–2014, and 2014–2023

The intensity analysis of the LULC dynamics in the study region is presented in Fig. 6. The results revealed varied dynamics of the LULC classes driven by anthropogenic forces due to mining operations in the region. The category level analysis (Fig. 6) shows the size and annual intensity of change of each category’s gain relative to the size of the category and the interval end time point. The plot shows that built-up, bareland, and agricultural land gained the most during all the time intervals. The result revealed that bareland, built-up areas, and agricultural land had active changes during the two intervals. The right side of Fig. 6a shows that the bar for gain of bareland, built-up areas, and agricultural land extends to the right of the uniform line in both time intervals except agricultural land in the first interval. In terms of loss, shrubland areas and forestland areas were the major losers (Fig. 6b). The right side of Fig. 6b the forested area extends to the right of the uniform rate during the first and the third period indicating that the intensity loss of the forest area was active during these periods. Similarly, the bar for loss of shrubland areas extends to the right of the uniform rate in the first and second intervals; however, the intensity of loss was more active during the second period (Fig. 6b). The gross changes of the LULC category revealed that the forest, shrubs, and grasses experienced net losses during the study period.

### Land use transitions

**Fig. 7:**
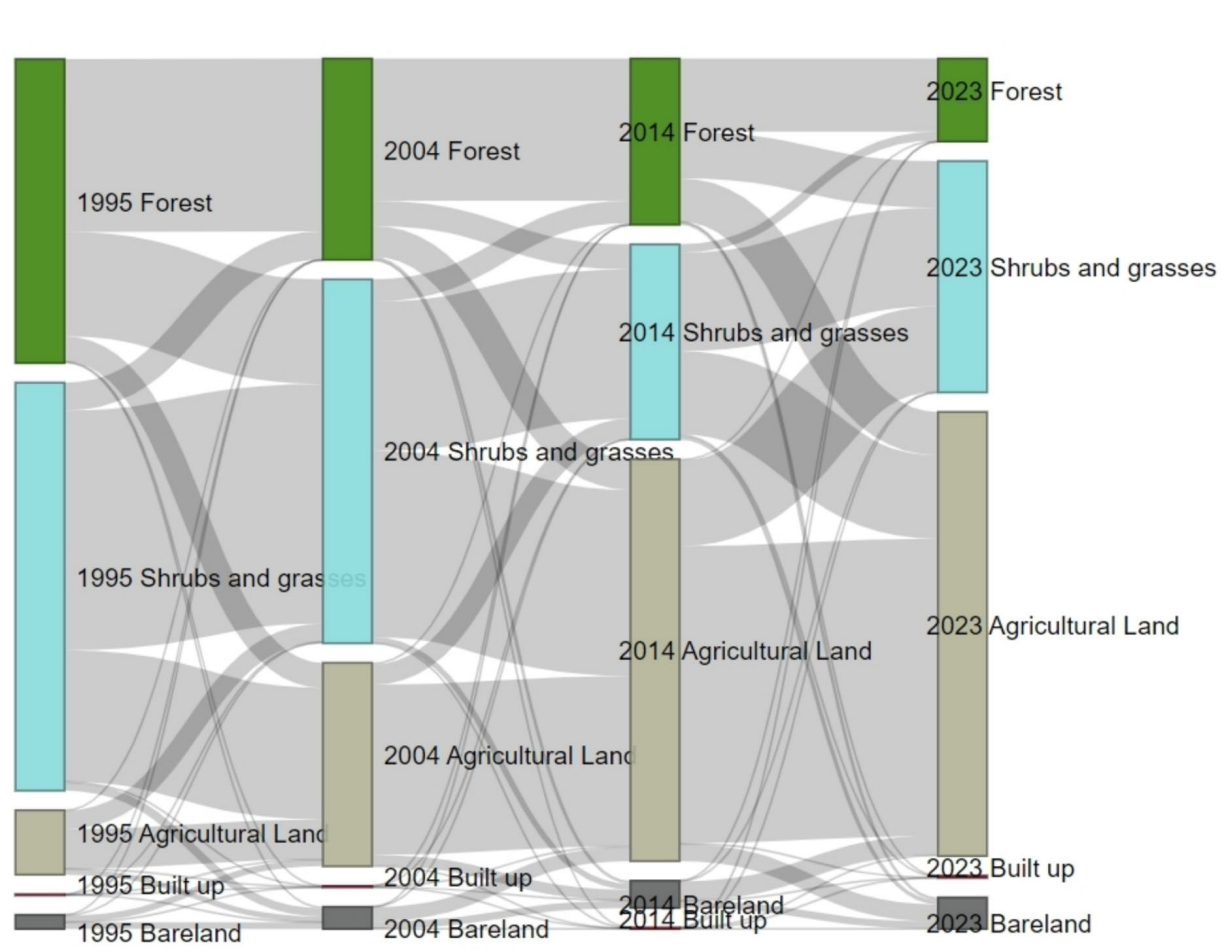
Sankey plot showing the LULC transitions between 1995, 2004, 2014, and 2023 in the study area

A Sankey plot (Fig. 7) illustrated the relative changes in the LULC classes of the study area over three distinct years: 1995, 2004, 2014, and 2023. The plot provides a robust visualization of transitions between different land cover types, highlighting how various LULC classes have evolved following the discovery and initiation of mining operations. It is evident from the results that much of the forestland in the first period transitioned to agricultural land and shrubs and grasses. A similar pattern was observed in the second period (Fig. 7). Shrubs and grasses in the first period lost much of their coverage to agricultural lands (82.57 km^2^), with a small portion converting to forestlands (9.86 km^2^).

In the second period, agricultural lands further expanded, but part of the gains from the first epoch (37.89 km^2^) were lost to shrublands. Nonetheless, agricultural lands maintained a steady growth across the two analyzed intervals. Forests reduced further in the second decade, losing most of its coverage to shrubs and grasses (20.74 km^2^) and agricultural land (19.25 km^2^). Similarly, bareland areas steadily increased in area over the two decades, gaining much of its coverage from shrubs and grasses, and forest (Fig. 7). Built-up areas expansion from 1995 to 2014 was slow; however, from 2014 to 2023, the expansion surged, reflecting rapid urbanization and infrastructural development in the region. This trend underscores ongoing urbanization due to mining activities as the result of the conversion of natural or semi-natural landscapes into urban areas.

### Perceived environmental, social, and economic implications of gold mining

#### Household socio-economic characteristics

Table 3 summarizes the socio-economic characteristics of the population in the study area. The survey revealed that most household heads in the study area were males, constituting about 69.9%, while 30.1% were females. Most respondents (48.2%) had attained primary-level education, while a few (9.6%) had no formal education. Most residents (38.6%) in the area engaged in farming, whereas 15.7% were involved in mining. The average age of the respondents was 37 years, while the mean household size in the study area was 6 (Table 3).

**Table 3:** Summary of the sampled households’ socio-economic characteristics

Variables	Characteristics	(*n* = 83)	(%)	Min	Max	Mean	Std. dev
Gender	Female	25	30.1				
	Male	58	69.9				
Age (years)		83		18	75	37.1	10.8
Marital status	Single	14	16.9				
	Married	61	73.5				
	Separated	6	7.2				
	Divorced	1	1.2				
	Widowed	1	1.2				
Education level	No formal education	8	9.6				
	Primary	40	48.2				
	Secondary	17	20.5				
	Tertiary	18	21.7				
Occupation	Skilled labor	4	4.8				
	Businessperson	9	10.8				
	Casual labor	13	15.7				
	Civil servant	12	14.5				
	Farmer	32	38.6				
	Miner	11	15.7				
Household size	Number of members	83		1	25	5.5	3.7

#### Perceived environmental, social, and economic implications of mining

**Fig. 8:**
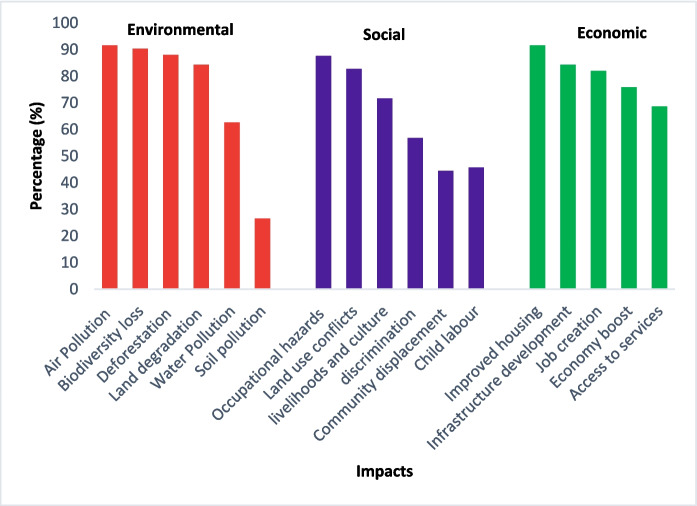
Perceived environmental, social, and economic impacts of mining in the study area

Linking the observed LULC changes from satellite images to the perceived social, economic, and environmental implications of the changes on the livelihoods of local communities is essential in LULC studies (Basommi et al., 2016a, 2016b). The majority of respondents acknowledged the impacts of mining in the study area, with 98% reporting social and economic implications, and 96% observing environmental consequences. The perceived environmental impacts of mining included air pollution (92%), biodiversity loss (90%), deforestation (88%), land degradation (84%), water pollution (63%), and soil pollution (27%). The top three social impacts of mining in the study area were occupational hazards, especially for the mine workers (88%), land use conflicts (83%), and negative impacts on livelihood and culture (72%). The leading perceived economic impacts of mining were improved housing (92%), infrastructure development (84%), and job creation (82%). The details of the perceived impacts of mining in the study area are presented in Fig. 8.

## Discussion

### LULC trends

The LULC analysis was performed with an overall accuracy exceeding 80% and a kappa coefficient greater than 0.68 (Table 1), which meets the recommended thresholds for LULC classifications. These accuracy levels surpass the classification accuracy assessment reported by Patil and Nataraja, (2020), reinforcing the reliability of the generated LULC maps. The LULC analysis and social survey results indicate that mining and associated activities are the key drivers of LULC dynamics in the Mang’onyi, Sambaru, and Londoni areas of the Singida region. This is because most of the changes occurred after the year 2004 when gold reserves exploration and mining intensified in the aforementioned villages (United Republic of Tanzania, 2014). While forests, shrubs, and grasses were the initial dominant land cover types in the study area in 1995 and 2004, respectively, agricultural land, bareland, and built-up areas became much more visible in 2014 and 2023 (Fig. 3). Built-up areas increased steadily over the study period due to general population growth and population influx into the study area after the discovery of gold reserves in 2004 (Lugoe, 2011; Ministry of Finance and Planning, 2022). The discovery of gold mineral reserves in the region enticed local artisanal miners, gold rush miners, and small-scale and large-scale mining companies to participate in mining operations (Jønsson & Bryceson, 2009). The influx of small business owners in the mining area also boosted the population density in the surrounding communities. This accelerated the construction of residences in mining camps and neighboring villages, thus increasing the build-up area over the study period (Fig. 4). The relocation of the inhabitants from the Shanta mining site to a newly planned and constructed village, particularly in the northern part of the study area, enhanced the visibility of the build-up region in 2023 (Fig. 3). Our findings align with findings from other studies (Basommi et al., 2016a, 2016b; Garai & Narayana, 2018; Gbedzi et al., 2022), which have documented similar trends of increasing built-up areas in the mining sites due to the mining-induced influx of people into the mining area, which created the demand for better housing.

A notable continuous forest decline (Table 2) was observed in the study area over the three decades (1995 to 2023). The forested area was significantly converted into shrublands, grasslands, agricultural fields, barelands, and built-up areas (Fig. 7). While the landscape underwent a significant and ongoing reduction in forest cover, other land cover type classes (agricultural land, bareland, and buildup) progressively expanded over the years. This pattern of forest change aligns with findings from other studies (Addo-Fordjour & Ankomah, 2017; Garai & Narayana, 2018; Kamga et al., 2020; Kumi et al., 2021), which have documented similar trends of declining forest cover, often transformed into other land use classes due to gold mining activities. The transformation of forested areas is linked to mining-related activities and other human-induced disturbances brought on by the influx of people to the mining site. This is evidenced by the population surge following the gold discovery in Londoni in 2004, where the village initially had approximately 1,600 subsistence farmers and livestock keepers, but within a few months, its population grew to over 10,000 as people engaged in hard rock gold mining (Jønsson & Bryceson, 2009). Mining operations generally require clearing vegetation (Mishra et al., 2022) to open up the mining site and provide lumber for the mining tunnels (open pit), leading to forest loss. The increase in population due to the influx of rush miners boosted demand for firewood and charcoal, exacerbating unlawful forest resource harvesting and thus decreasing forested areas over time. Additionally, mining activities in areas where farmlands existed led to the shifting of farming activities into the nearby forest zones. The intensification of agricultural operations towards the adjoining forest zone due to the great demand for food among the surrounding populations resulted in a decline in the forest areas. A study in Western Ghana similarly showed a substantial loss of farmlands within mining areas and widespread spill-over effects as displaced farmers expand farmland into forest areas (Schueler et al., 2011).

Interestingly, there was a constant rise in agricultural acreage (Table 2 and Fig. 7) over the years in the study area. The expansion of agricultural land in the study area is likely driven by the rising demand for food, spurred by population growth resulting from the influx of people attracted by mining activities. This increased population places additional pressure on land resources, necessitating the conversion of natural landscapes into agricultural fields to meet the growing food requirements. This aligns with the observations from other studies (Garai & Narayana, 2018; Kanianska, 2016; Kumi et al., 2021) that reported human activities significantly accelerate land-use change, driven by rapid population growth and the escalating demand for food to sustain the growing population. Changes in economic priorities (from mining to agriculture) among gold rush miners and population growth might explain the steady increase in agricultural land in the study area. This converted forest, bareland, shrub, and grassland areas into agricultural fields (Fig. 7). This is seen in the increase of agricultural fields in the Chipinga region in 2023 (Fig. 4), as most of the residents of Londoni Village established new farms in the Chipinga area.

Bareland areas, which are predominantly created through mining and human activities, similarly kept increasing throughout the study period since mining operations in the study area primarily involve surface (alluvial mining) and hard rock gold mining (underground mining tunnels) via excavation methods (Jønsson & Bryceson, 2009). Surface mining involves the removal of ground vegetation and soils, resulting in bareland (Schueler et al., 2011). Therefore, the continuous expansion of bareland can be attributed to the expansion of small-scale mining sites, large-scale production of gold by commercial companies, and the construction of roads for gold and agricultural products transportation within and out of the study area.

The changes in grasses and shrubs over time can be linked to conversion to other land cover types, such as bareland and agricultural (Gbedzi et al., 2022). Between 2004 and 2014, the incoming population converted many of the shrubs and grasses into agricultural lands to cater to their food production needs. Mining in the forest also led to forest degradation and conversion of forests to grass and shrubs. After the exhaustion of minerals in most of the mining areas between 2014 and 2023, the bareland was abandoned, allowing shrubs and grasses to grow and subsequently expand in area coverage.

### Environmental and socio-economic implications

Results from the social survey of the study area residents backed by relevant existing literature suggest that gold mining activities and the resultant LULC changes in the Singida region of central Tanzania have had significant environmental and socio-economic implications.

#### Environmental implications

The social survey established water and soil pollution as a key environmental impact of mining in the study area (Fig. 8). This finding is validated by a study carried out around the small-scale mines of Londoni and Sambaru, which revealed heavy metals (Hg, Pb, Zn, and Cu) contamination of water and soils above the permissible maximum Tanzanian limits (Herman & Kihampa, 2015). Sources of the heavy metal contamination were from mining-related activities including discharge of mine water to the surroundings, amalgamation and burning activities, improper waste rocks and tailings disposal, and leachates from waste rocks and tailings (Herman & Kihampa, 2015). The respondents also listed land degradation, deforestation, and biodiversity loss as environmental impacts of mining (Fig. 8). Surface mining interrupts ecosystem service flows, removes ground vegetation and soils, often causes irreversible loss of farmlands (Schueler et al., 2011). Surface mining also causes deforestation and habitat loss, which harms native wildlife and biodiversity. Clearing vegetation on the mining sites promotes soil erosion, which can further deteriorate the land. These findings align with that of Mencho (2022), who reported water pollution, deforestation, land degradation, and biodiversity loss due to gold mining operations in the Shekiso district, Guji zone, Ethiopia.

Air pollution, primarily generated by mine blasts and the resulting dust, is a major environmental concern in the region, particularly near the Shanta mining site. Many respondents voiced concerns about the spread of dust particles in the air following each blast, particularly noting its impact on visibility for drivers near the site. The dust becomes so thick that drivers are often forced to turn on their headlights even during the day to improve visibility and ensure safer driving conditions. Additionally, residents reported multiple instances of bird deaths and foxes around the Shanta mining site, suspecting that the birds drank untreated, uncovered wastewater. This can pose a significant health risk to children who may collect and consume these birds when their parents are not at home. Without proper supervision, exposure to contaminated birds could pose significant health risks, including cancer, as these toxic chemicals can accumulate and transfer to higher trophic levels. This underscores the urgent need for immediate action to mitigate this issue. In Sambaru, near the Yusuph Mwandami vat leaching plant, residents reported the deaths of 11 cows in 2022 and two more in 2023, attributing these fatalities to the consumption of wastewater from gold processing operations. Several cases of goats, sheep, and other animals dying have been reported due to insufficient fencing around the mining areas, which allows animals to access the wastewater sites. This lack of proper barriers has led to the animals’ exposure to hazardous waste, resulting in their deaths and eventually leading to biodiversity loss. The study underscores that the environmental impact of these mining operations in the area is severe and must be addressed immediately.

#### Social implications

Occupational hazards for mine workers in the form of exposure to dust, chemicals, and unsafe working conditions were listed as a key social impact in the study region (Fig. 8). Results from a similar study carried out at Londoni village in the Manyoni District of Singida region revealed that mineworkers were exposed to health and safety risks due to a lack of protective gears and rudimentary technologies used in gold extraction (Ringo & Kingu, 2018). These risks lead to diseases (diarrhea, flu, and backbone pains), injuries, loss of lives, and property loss due to exposure to chemicals and machine accidents. Negative impacts on local livelihoods and the culture of residents were also attributed to the mining activities. Ringo and Kingu (2018) established social vices resulting from the existence of mining activities in the study area. According to Ringo and Kingu (2018), there has been a notable rise in sexually transmitted diseases (STDs) from unsafe sexual intercourse and increased drug and substance abuse in and around the mining camps. Some respondents, particularly those with high blood pressure, voiced concerns about the noise pollution caused by blasting rock minerals, especially around the Shanta mining site. In some cases, individuals even collapsed due to the extreme noise levels during the blasts. The problem is more severe for individuals with critical conditions, who are notified of blasting days in advance and evacuated to safer areas away from the blasting site. This highlights the severity of the issue, as the high-pitched noise from the blasting poses serious health risks, particularly for those with pre-existing conditions.

Community displacement and land use conflicts were other outstanding social impacts of mining in the area. Although the intention of evacuating the surrounding community, particularly near the Shanta mining site, was well-meaning, respondents still expressed concerns about the lack of essential services such as access to clean water, hospitals, and schools in their new settlement. The absence of these critical services has left the displaced communities struggling to meet these services despite the evacuation efforts. The inadequate compensation and lack of public education about the terms and pricing of land compensation have intensified land use conflicts between the Shanta mining company and the surrounding community. This lack of clarity and fair compensation has increased tensions and disputes over land ownership and usage. Previous studies have likewise reported land use conflicts between miners due to the large operation areas demand, and the surrounding communities who depend largely upon the land for their livelihoods (Hilson, 2002; Schueler et al., 2011). Child labor and discrimination of women in the allocation of mining jobs were also mentioned as other social impacts. Child labor is common in the small-scale gold mines in Tanzania. Household poverty is the main factor pushing children to work in the mines due to their households’ inability to provide for their basic needs (Metta et al., 2023). The unequal participation of males and females in mining activities has a social impact contributing to financial disparities among household members. Our study aligns with the study conducted in the Prestea–Huni Valley Municipality of Ghana, which reported that women involved in artisanal gold mining were correspondingly discriminated against via cultural marginalization, poor work support services for women with children, poor working environment, and inter-ethnic discrimination by employers (Arthur-Holmes & Abrefa Busia, 2021).

#### Economic implications

The economic implications of gold mining were mostly positive as they comprised improved housing, infrastructural development, job creation, and improved access to social services. Gold mining and processing activities contribute to the economic development of the study region and Tanzania as a whole since it offers employment and an alternative source of income to the mine workers (Merket, 2018; Phillips et al., 2001; Ringo & Kingu, 2018). Respondents reported experiencing economic growth following the discovery of gold deposits in the area in 2004, which provided an alternative source of income and significantly improved their livelihoods. This newly discovered resource led to greater financial stability and opportunities for the local community, as demonstrated by improved housing conditions (Fig. 8). The influx of gold miners, mining companies, mine workers, and business people in the area created a heightened demand for better housing. This influx of people significantly increased the need for improved living accommodations to support the growing population. Infrastructure improvements throughout the year have significantly boosted the import and export of goods, particularly benefiting small business owners and farmers, resulting in better trade and economic opportunities. The increasing population also led to a higher demand for food, which boosted agricultural activities, such as irrigation practices in nearby villages, ultimately improving the livelihoods of the surrounding community. This rise in agricultural engagement provided greater food security and enhanced economic opportunities for residents. This finding is echoed by Haundi et al. (2021) who assert that artisanal and small-scale gold mining are key sources of informal employment for local communities and an alternative source of income for rural farmers, especially during the dry season when farming becomes unfavorable. A study by Pokorny et al. (2019) in Burkina Faso likewise associated better wages, infrastructural development, improved social programs, and new business opportunities with mining operations.

## Conclusions

This study demonstrates that gold mining has been a significant driver of LULC changes in the Singida region, particularly in the Mang’onyi, Sambaru, and Londoni mining sites within the Ikungi and Manyoni districts. Over the period from 1995 to 2023, mining activities contributed to the expansion of agricultural land, bareland, and built-up areas, while forest cover, shrublands, and grasslands have declined. These findings suggest that mining-induced land transformations could have long-term environmental consequences, including habitat degradation, soil erosion, and water contamination. Beyond the environmental impacts, the study further reveals socio-economic challenges faced by local communities, such as land displacement, loss of traditional livelihoods, and conflicts over resource use. Community perceptions highlight concerns about livelihood sustainability, inadequate compensation mechanisms, and environmental degradation, emphasizing the urgent need for integrated land management policies.

While mining has economic benefits, its sustainability remains in question if environmental regulations and rehabilitation measures are not adequately enforced. Therefore, policymakers, mining companies, and local communities must collaborate to establish responsible mining practices. This includes enforcing stronger environmental regulations, implementing land restoration programs, improving compensation mechanisms, and integrating local communities into decision-making processes. Balancing economic gains from gold mining with environmental conservation and social equity is imperative. Without proactive interventions, the long-term sustainability of land resources and community well-being in the study area could be compromised. This study underscores the need for science-driven policy interventions to ensure that mining remains a catalyst for development without jeopardizing ecosystem health and local livelihoods. The study acknowledges limitations such as the need for long-term monitoring to capture finer-scale land changes and their cascading effects. Future research should explore quantitative assessments of soil and water quality, biodiversity losses, and socio-economic livelihood transitions to provide a more comprehensive understanding of mining’s long-term effects.

## Data Availability

No datasets were generated or analysed during the current study.
